# Impact of *Moringa oleifera* Leaf Powder Addition on the Properties of Cranberry‑Based Gummies and Marshmallows With Low‐Glycemic Sweeteners

**DOI:** 10.1111/1750-3841.71322

**Published:** 2026-07-28

**Authors:** Lourdes Cervera‐Chiner, María Luisa Castelló, Francisco José García‐Mares, María Dolores Ortolá

**Affiliations:** ^1^ Food Engineering Research Institute—FoodUPV Universitat Politècnica de València Valencia Spain; ^2^ Department of Hydraulic Engineering and Environment Universitat Politècnica de València Valencia Spain

## Abstract

Traditional sweets are associated with dental caries, high blood glucose, and obesity. This study evaluated the impact of incorporating *Moringa oleifera* leaf powder (MOLP) into soft candies (gummies and marshmallows) formulated with a fixed base matrix of cranberry juice and low‐glycemic, noncariogenic sweeteners (isomaltulose, oligofructose, and tagatose). MOLP was incorporated at 0%, 2.5%, 5.0%, and 7.5% while keeping the proportions of cranberry juice and sweeteners constant, enabling isolation of the specific contribution of MOLP. The increase in MOLP levels produced statistically significant increases in the antioxidant capacity and phenolic content of the candies, which confirms their functional potential. MOLP addition also produced statistically significant changes in color, shifting products toward darker, greenish‑yellow tones due to moringa pigments. Texture was matrix‑dependent: Gummies became progressively firmer and chewier with increasing MOLP, whereas marshmallows presented moderate structural changes attributable to their aerated nature. Sensory evaluation revealed the highest consumer acceptance for the control and 2.5% formulations, with a reduced liking at 7.5% due to bitterness, color changes, and excessive firmness. Purchase intent remained positive when health benefits were communicated. Overall, moderate MOLP inclusion (2.5%–5%) provides a practical balance between functional enhancement and sensory appeal for cranberry‑based soft candies formulated with low‑glycemic sweeteners.

**Practical Applications:**

This study shows that gummies and marshmallows can be made with low‐glycemic sweeteners and plant ingredients such as moringa to improve their nutritional quality. The results can help food producers develop sweets with added antioxidant value while keeping acceptable taste and texture. These products may be of interest to consumers seeking confectionery options with a more health‐oriented composition.

## 1 Introduction

Gummy candies are a type of food that both children and adults enjoy for pleasure. On average, the consumption of sweets per person in Spain is 24 kg per year (Sadival 2019). In Spain, gummy candies are very popular and represent a large share of confectionery consumption, accounting for 52% of the total consumption. They are followed by hard candies, which make up 30%, while chewing gum represents the remaining 18% (Interbenavente 2023). Spain is a strong player in this sector, with large business volumes in both imports and exports. The confectionery industry in Spain is booming, with sales exceeding 7.5 billion euros in 2023, highlighting the high demand for sweet products, including gummy candies (Interbenavente 2023). Among the trends in the sector, progressive digitalization is evident, reflected in the growth of online sales. Another long‐standing trend is the growing interest in sugar‐free gummy candies, hard candies, and other sweet snacks. A report by Global Research Consulting predicts an annual growth rate of 5.3% in the consumption of these products between 2021 and 2028 (Interbenavente 2023). This range of products is considered a healthier alternative to traditional sugar‐based confectionery. It helps reduce the risk of cavities and tooth erosion and contributes to a healthier diet by avoiding diseases associated with sugar consumption, such as obesity, diabetes, and cardiovascular diseases.

Conventional soft candies (gummies and marshmallows) are typically formulated with high‑glycemic sugars (e.g., sucrose, glucose syrup) that contribute to rapid postprandial glycemic responses, dental caries, and excessive caloric intake (Teixeira‐Lemos et al. 2021). In parallel, their matrices are often poor in bioactive compounds and dietary fiber, offering limited nutritional value. These issues are particularly relevant for children and consumers with impaired glucose regulation or weight‑management needs, as well as for broader public health goals targeting sugar reduction in confectionery. Replacing sucrose with isomaltulose, oligofructose, and tagatose has been shown to lower cariogenic potential and attenuate the glycemic response while maintaining sweetness and processability in confectionery systems (Periche et al. 2014, 2015). Isomaltulose is a natural disaccharide composed of glucose and fructose, like regular sugars, but linked by a stronger glycosidic bond of the α‐(1→6) type, which cannot be broken down by the bacteria responsible for dental cavities (Shyam et al. 2018). Therefore, it is noncariogenic and is slowly released into the bloodstream (De Souza et al. 2022). Oligofructose, on the other hand, is a natural sweetener derived from chicory and is composed of short chains of fructose and glucose. It has properties similar to those of sugar but provides fiber, making it a good sugar substitute. Furthermore, oligofructose is a prebiotic, as it has been found to promote the growth of intestinal microbiota strains such as *Lactobacillus lactis*, *Lactococcus lactis*, and *Saccharomyces boulardii*, thus promoting health (Pranckute et al. 2014; Kolida et al. 2002). Tagatose is a monosaccharide found in dairy products and can be industrially produced from lactose after it is hydrolyzed into glucose and galactose (Roy et al. 2018). It has hypoglycemic effects, can lower blood sugar levels, and helps regulate insulin and glucose metabolism, making it suitable for individuals with diabetes. Moreover, it also promotes the growth of beneficial gut bacteria. Its taste is as sweet as that of sucrose, but it has fewer calories, specifically, 1.5 kcal/g of tagatose (Roy et al. 2018). Previous research on jellies, marmalades, and marshmallows (Benucci et al. 2024; Rubio‐Arraez et al. 2018, 2016) demonstrated feasibility and quality improvements (e.g., stability, texture, functional composition, and sensory acceptability) when these sweeteners replaced sucrose/glucose syrups, supporting their use as healthier alternatives.

In the present study, the reformulated confectionery matrix was not only based on low‐glycemic sweeteners but also incorporated cranberry juice as a functional ingredient. Therefore, beyond the choice of sweeteners, it is important to consider the contribution of the fruit matrix itself to the nutritional and technological characteristics of the final products. Cranberry (*Vaccinium macrocarpon*) juice was selected as the base fruit matrix of the formulations evaluated in this study. Beyond its contribution to flavor and color, cranberry juice is rich in polyphenols (e.g., proanthocyanidins, anthocyanins) and possesses notable antioxidant properties. Therefore, its incorporation may contribute to improving the functional profile of confectionery products while simultaneously providing a natural source of antioxidants measurable by DPPH and electrochemical methods, thus increasing the bioactive content of the final product. Integrating cranberry juice into the base matrix allows nutritional enhancement without the use of added synthetic colorants while its acidity helps control the pH within ranges unfavorable to pathogenic bacteria (Kalin et al. 2015).

While cranberry juice constituted the fixed fruit matrix of the formulations, *Moringa oleifera* leaf powder (MOLP) was incorporated as the novel functional ingredient whose effects were systematically investigated in this study. Moreover, MOLP is a concentrated source of phenolics, vitamins, minerals, essential amino acids, and fiber (Arora and Arora 2021; Sultana 2020) with documented antioxidant and potential health‑promoting effects (Wang et al. 2024; Farooq et al. 2012). Its incorporation into food matrices (bakery, snacks) has demonstrated gains in antioxidant capacity and phenolic content, but its impact on soft‐candy texture, color, and consumer acceptance of low‐glycemic formulations remains insufficiently characterized (Trigo et al. 2023; Ma et al. 2020; Chinchilla et al. 2020; Cervera‐Chiner et al. 2024). Although moringa‐based gummies are commercially available, their presence in the global market appears to be limited, and many existing formulations reported in the literature rely on high‐sugar matrices typical of conventional gummy products. This study was designed to isolate the effects of MOLP on soft candies by keeping the cranberry juice and low‐glycemic sweetener matrix constant (cranberry juice, isomaltulose, oligofructose, and tagatose at fixed proportions) throughout all formulations. MOLP was the only experimental factor that varied (0%, 2.5%, 5.0%, and 7.5%), and its impact on the antioxidant/phenolic content, color, texture, and consumer acceptance was quantified in gummies and marshmallows.

Unlike prior studies that focused on substituting sucrose with individual healthy sweeteners or on general marshmallow/gummy reformulations, our study integrates three low‑glycemic sweeteners with cranberry juice as a constant base matrix and systematically varies the MOLP as the sole factor, enabling a clear attribution of functional and sensory changes to the MOLP under a controlled healthier confectionery framework. In addition, we combined instrumental analyses (texture profile analysis [TPA], color, a_w_, °Brix, pH) with consumer sensory evaluation, Just‐About‐Right (JAR) scale, and penalty evaluations, identifying optimal MOLP ranges that balance functional improvements with consumer acceptance, findings that can be directly applied to new product development.

Therefore, the objective of this study was to develop and characterize a healthy alternative to traditional soft candies (marshmallows and gummies) by using functional ingredients such as MOLP, cranberry juice, and low‐glycemic and noncariogenic sugars (isomaltulose, oligofructose, and tagatose). The key technological challenge is balancing healthier compositions (low‑glycemic sweeteners, added bioactive compounds from cranberry juice and MOLP) with desirable textures and sensory acceptance. Elevated MOLP levels may increase hardness/chewiness and alter color, potentially penalizing liking; conversely, insufficient MOLP levels may underdeliver functional benefits. Therefore, in the present work, the effects of MOLP addition (0%–7.5%) on a fixed base of cranberry juice and low‐glycemic sweeteners were isolated, and the impacts on physicochemical properties, antioxidant capacity/total phenolics, instrumental texture, and sensory acceptance in gummies and marshmallows were quantified.

## 2 Materials and Methods

### 2.1 Moringa Leaf Powder Production

MOLP was obtained from moringa leaves collected from trees in the experimental plot at the Universitat Politècnica de València (Spain), which is located at 39°29'00.5“N 0°20'04.1”W. The leaves were cleaned with water and dried at room temperature (22°C) overnight. After that, they were dried at 50°C for 11 h in a convective tray oven (CLK 750 TOP+, POL‐EKO, Wodzisław, Poland). The leaves were subsequently depeciolated and crushed at 10,200 rpm via a food processor (Thermomix TM31 Vorwerk, Wupertal, Germany). The powder was stored in airtight glass jars in the dark at room temperature until the formulations were prepared.

### 2.2 Soft Candy Formulation and Preparation

Two types of soft candies were made from the same syrup. One of them had an airy texture (marshmallows), and the other had a chewy texture (gummies).

To unambiguously attribute changes to MOLP, the base matrix was held constant across all formulations (cranberry juice 36%, isomaltulose 19%, oligofructose 20%, tagatose 20%, gelatin 5%), and only the MOLP content was varied (0%–7.5%), proportionally replacing the remaining components while preserving their relative ratios. Thus, the effects of cranberry juice and sweeteners were not studied as substitutes; instead, they provide a fixed background to evaluate the technological and sensory consequences of MOLP addition.

The control syrup for the candies (without MOLP) was prepared with three noncariogenic sweeteners with a low glycemic index: 19% isomaltulose (Palatinose powder VitoBest; Alicante, Spain), 20% chicory oligofructose (Moara, Foody; Madrid, Spain), 20% tagatose (Tagatesse, Damhert Nutrition; Rijssen, Netherlands), 36% commercial cranberry juice (Hacendado, Madrid, Spain), and 5% gelatin A 220 Bloom (Junca Gelatines S.L.; Girona, Spain). The proportions of the different sweeteners used were optimized in previous unpublished work by this research group.

Additionally, different syrups were prepared by adding MOLP in varying proportions (2.5%, 5%, and 7.5%) to the control formulation. Notably, adding a percentage of MOLP greater than 7.5% was not possible because the mixture became solid, and it was not feasible to make the gummies, according to some previous trials (data not shown).

The formulation of the syrups with MOLP was carried out by proportionally replacing the rest of the components with the percentage of MOLP incorporated. A total of eight different formulations were developed: four marshmallow‐type formulations (M0, M2.5, M5, and M7.5) and four gummy‐type formulations (G0, G2.5, G5, and G7.5). Table 1 shows the amounts of each component in the formulations for 100 g of syrup.

**TABLE 1:** Percentage of ingredients for each soft candy formulation.

Formulation	Isomaltulose	Oligofructose	Tagatose	Cranberry juice	Gelatin	MOLP
M0/G0	19	20	20	36	5	0
M2.5/G2.5	18.53	19.5	19.5	35.10	4.88	2.5
M5/G5	18.05	19.0	19.0	34.20	4.75	5.0
M7.5/G7.5	17.58	18.5	18.5	33.30	4.63	7.5

*Note*: M refers to marshmallow‐type candies, and G refers to gummy‐type candies.

Five hundred grams of syrup was prepared using a food processor (Thermomix, TM31, Vorwerk, Wuppertal, Germany) for each formulation. In the case of gummy‐type candies, gelatin was first dissolved at a 1:2 (w/v) ratio with hot cranberry juice and then set aside. Moreover, the sugars, moringa leaf powder, and remaining juice were stirred at 200 rpm and heated for 5 min at boiling temperature. After that, the dissolved gelatin was added, and the mixture was again stirred at 200 rpm for 5 min at 60°C. The resulting mixture was then poured into cylindrical silicone molds, which were 28 mm in diameter and 20 mm in height (Silikomart SCG07), previously greased with sunflower oil (Koipesol, Seville, Spain), and they were left to rest for 24 h at room temperature to solidify.

For the preparation of the marshmallow‐type gummies, the same syrups used for the gummy‐type formulations were employed, following the same steps described above. However, after the gelatin was added to the sugar, cranberry juice, and MOLP mixture, the food processor was set at 2000 rpm for 10 min at 60°C to aerate the syrup and achieve the characteristic marshmallow texture. The mixture was then distributed into silicone molds using a piping bag and left to solidify for 24 h at room temperature.

The gummies and marshmallows were subsequently removed from the molds and stored in glass containers at room temperature (22°C) until further analysis. These formulations were made in triplicate.

### 2.3 Experimental Design

The experimental design was based on a single‐factor formulation approach in which the percentage of MOLP was the independent variable. Four MOLP levels were evaluated: 0%, 2.5%, 5.0%, and 7.5% (w/w). Two confectionery matrices were produced from the same base syrup: gummies and marshmallows. The base formulation consisted of cranberry juice, isomaltulose, oligofructose, tagatose, and gelatin, which were maintained at constant relative proportions throughout the study. MOLP was incorporated by proportionally replacing the remaining components while preserving the matrix composition.

The dependent variables evaluated were moisture content, water activity (a_w_), °Brix, pH, color coordinates (L*, a*, b*, C*, h°), total color difference (ΔE), antioxidant capacity, total phenolic content, texture profile parameters, sensory acceptability, JAR responses, and purchase intent.

All formulations were produced in triplicate. Physicochemical analyses were performed in triplicate, color and texture analyses were conducted with six replicates, and sensory evaluation involved 60 consumers.

### 2.4 Analytical Determinations

#### 2.4.1 Physicochemical Analysis

The moisture content and water activity of the final products were analyzed. The moisture content was determined using a gravimetric method, where samples were dried to a constant weight in a vacuum oven at 60°C (AOAC 2000). Water activity (a_w_) was measured using a dew point hygrometer (FA‐st lab, GBX, Valence, France). The soluble solid content (°Brix) was assessed with a refractometer at 20°C (ATAGO 3T, Tokyo, Japan), whereas the pH of the initial syrup was measured using a pH meter (SevenEasy, Mettler Toledo, Greifensee, Switzerland).

#### 2.4.2 Color

The color of each formulation was measured using a Konica‐Minolta spectrophotometer (model CM‐3600d, Singapore, Republic of Singapore) by placing the sample on the diaphragm aperture (8 mm). The CIEL*a*b* coordinates were obtained using the D65 illuminant and a standard observer (10° visual field) as references. The registered parameters were L*(brightness), a* (red component), b* (yellow component), C* (chroma), and h° (hue angle).

In addition, the color differences (ΔE) of each candy formulation were calculated using Equation (1) with respect to control candies.

(1) $$\Delta E^{*}=\sqrt{{\left(\Delta a^{*}\right)}^{2}+{\left(\Delta b^{*}\right)}^{2}+{\left(\Delta L^{*}\right)}^{2}}\;$$

#### 2.4.3 Antioxidant Capacity and Total Phenol Content

The antioxidant capacity of the gummies/marshmallows was determined via two methods: the 2,2‐diphenyl‐1‐picrylhydrazyl (DPPH) free radical scavenging activity method and the electrochemical antioxidant method.

##### 2.4.3.1 DPPH Method

The DPPH method is based on the scavenging capacity of the stable free radical DPPH, and the protocol described by Periche et al. (2016) was followed, with some modifications. One gram of each candy was weighed, and 10 mL of a methanol:water (80:20) solution (v/v) was added. The mixture was homogenized with a blender (IKA T‐25 Utra‐Turrax; Wilmington, USA) for 1 min. Then, the mixture was shaken for 10 min with a magnetic stirrer and subsequently centrifuged at 10,000 rpm for 5 min in a centrifuge (Eppendorf centrifuge 5804R; Hamburg, Germany). Next, 0.1 mL of this extract was mixed with 3.9 mL of 0.025 mg/mL methanolic solution of DPPH (purity > 95%, Sigma‒Aldrich). The mixture was incubated for 30 min in the dark, and then the absorbance was measured at 515 nm with a spectrophotometer (Thermo Fisher Scientific, Inc., Helios Zeta UV–VIS, Waltham, MA, USA). The DPPH solution was used as the reference absorbance. The quantification was performed considering a standard curve of Trolox (6‐hydroxy‐2,5,7,8‐tetramethylchroman‐2 carboxylic acid, purity > 98%, Sigma–Aldrich) with concentrations between 25 and 750 µM. The results were expressed as mg of Trolox equivalent (TE) per 100 g of candy.

##### 2.4.3.2 Electrochemical Antioxidant Activity Determination

The antioxidant capacity was also measured with a BRS device (BQC Redox Technologies, Oviedo Asturias, Spain), an electrochemical instrument specifically designed for rapid, precise, and straightforward assessment of the redox state in diverse samples (Capaldi et al. 2024; Postigo et al. 2023; Corpas and Palma 2024). The system is based on the measurement of redox potential (charge/period or micro‐Coulomb, µC). The same extracts prepared via the DPPH method were diluted in distilled water in a proportion (1:8, v/v) to reduce the percentage of methanol to the requirements of the BRS method (< 10%), and then 100 µL of this mixture was mixed with 100 µL of the electrolyte solution from BQC. After that, 50 µL of this mixture was placed in the test area of ​​the BRS strip, and the measurement was performed. The measurements were expressed in microcoulombs (µC) and converted to TEs via a calibration curve integrated within the instrument.

##### 2.4.3.3 Total Phenolic Content

The total phenolic content was determined using the spectrophotometric Folin–Ciocalteu method, as described by Singleton et al. (1999). For the extraction of phenolic compounds from the gummies, samples were diluted 1:10 (w/v) with 80% methanol and subsequently centrifuged at 13,000 rpm for 5 min. In the case of moringa leaf powder, 1 g of sample was processed following the same extraction procedure. After centrifugation, the obtained supernatant was further diluted 1:10 with distilled water prior to analysis. For cranberry juice, a 1:10 dilution with distilled water was first prepared; subsequently, 1 mL of this dilution was mixed with 10 mL of 80% methanol and processed in the same manner as the gummy samples. After centrifugation, 0.125 mL of the supernatant of these extracts was transferred to a spectrophotometer cuvette and mixed with 0.5 mL of distilled water and 0.125 mL of Folin–Ciocalteu reagent (Sigma–Aldrich). The reaction mixture was allowed to stand in the dark for 6 min, after which the reaction was stopped by adding 1.25 mL of 7% (w/v) Na_2_CO_3_ prepared in distilled water, followed by the addition of 1.0 mL of distilled water. The mixture was incubated in the dark for 90 min, and the absorbance was then measured at 760 nm via a Helios Zeta UV/Vis spectrophotometer (Thermo Scientific). The results are expressed as milligrams of gallic acid equivalents per 100 g of sample (mg GAE/100 g) based on a gallic acid calibration curve in the concentration range of 0–700 ppm.

#### 2.4.4 Texture Profile

A TPA test was conducted to objectively determine the texture of the samples through six parameters. The methodology described by Periche et al. (2014) was employed with slight modifications. For this, a TA. XT plus texture analyzer (Stable Micro Systems, Godalming, UK) equipped with a 50 kg load cell and a 60.5 mm diameter cylindrical probe was used. The test consisted of applying 50% double compression to the sample with a 15‐s interval between cycles. The test speed was 1 mm/s.

The TPA test is based on recognizing texture as a multiparametric attribute. From the resulting force‒time curve, the following parameters were obtained, as defined by Bourne (1978): hardness (N), the maximum force during the first compression cycle; springiness, the height recovered by the sample during the time between the end of the first cycle and the beginning of the second cycle; adhesiveness, the negative force area for the first bite and representing the work required to overcome the attractive forces between the surface of a food and the surface of other materials with which the food comes into contact; chewiness, the energy required to masticate a solid food, calculated as the product of hardness × cohesiveness × springiness; cohesiveness, the ratio of the positive force area during the second compression to that of the first compression; and resilience, the ability of the sample to recover from deformation in terms of velocity and derived forces.

### 2.5 Sensorial Analysis

A total of 60 adult consumers (32 women, 28 men; 18–60 years of age) were recruited via open calls from university staff/students and the local community. The inclusion criteria were habitual confectionery consumption (≥ 1–2 times/week) and no self‐reported taste/smell disorders. The exclusion criteria were allergies/intolerances to gelatin, cranberry, moringa, or the study sweeteners; ongoing dental treatment limiting chewing; or dietary restrictions incompatible with tasting sweets. All participants provided informed consent.

Sensory evaluation was conducted in a tasting room at the Food‐UPV Institute, standardized according to the ISO 4121:2003 (ISO 4121:2003 2003) and UNE‐87025:1996 (European Standards 1996) standards. The tasting session was carried out by presenting four marshmallow‐type or four gummy‐type samples on different plates, which were covered with plastic cups to avoid influencing the tasters. The samples were coded with randomly assigned three‐digit numbers, and the sample presentation order was randomized and balanced across participants to reduce order and carry‐over effects. The questionnaires were administered through the Microsoft Forms platform (Office 365), where participants responded to a hedonic scale evaluation assessing the following attributes: overall appearance, color, aroma, texture, hardness, chewiness, flavor, sweetness, and overall acceptance, using a 9‐point scale (1 = “*dislike extremely*” and 9 = “*like extremely*”).

For the attributes of color, aroma, hardness, chewiness, flavor, and sweetness, the perceived intensity was assessed using a JAR scale. JAR scale is widely used in product optimization studies to determine whether the intensity of specific attributes is perceived as insufficient, excessive, or close to the ideal level from the consumer perspective.

Furthermore, a penalty analysis was performed to determine whether an attribute above or below the ideal point negatively affected the overall acceptance score (Ares et al. 2017; Li et al. 2014). Finally, a purchase intent question was included along with an additional question about purchase intent that considered the health benefits of the gummies/marshmallows. The wording of this last question was “If you knew that this product was healthier than the traditional product because it was made with low glycemic index sugars, therefore having a lower calorie count, not causing cavities, and possessing antioxidant and prebiotic properties, would you be willing to buy it instead of the one you usually buy?”

#### 2.5.1 Ethical Approval for Sensory Analysis

The study was reviewed and approved by the Research Ethics Committee of the Universitat Politècnica de València, under Authorization Number P04_30‐01‐2026, and informed consent was obtained from each participant prior to their participation in the study.

### 2.6 Statistical Analysis

Unless otherwise stated, physicochemical assays (moisture, a_w_, °Brix, pH) were performed in triplicate per formulation; color measurements in *n* = 6 replicates; TPA in *n* = 6 replicates per sample; and sensory evaluation with *n* = 60 adult consumers. The results are reported as the means ± SDs. The statistical analysis of the results was carried out using the Statgraphics Centurion software, version 19.1.2 (2019). A one‐way (ANOVA) parametric test was performed to determine the effect of the percentage of MOLP added to the final product, with a confidence level of 95%. Furthermore, principal component analysis (PCA) was applied to describe the relationships between the sensory and instrumental texture measurements.

## 3 Results and Discussion

**FIGURE 1:**
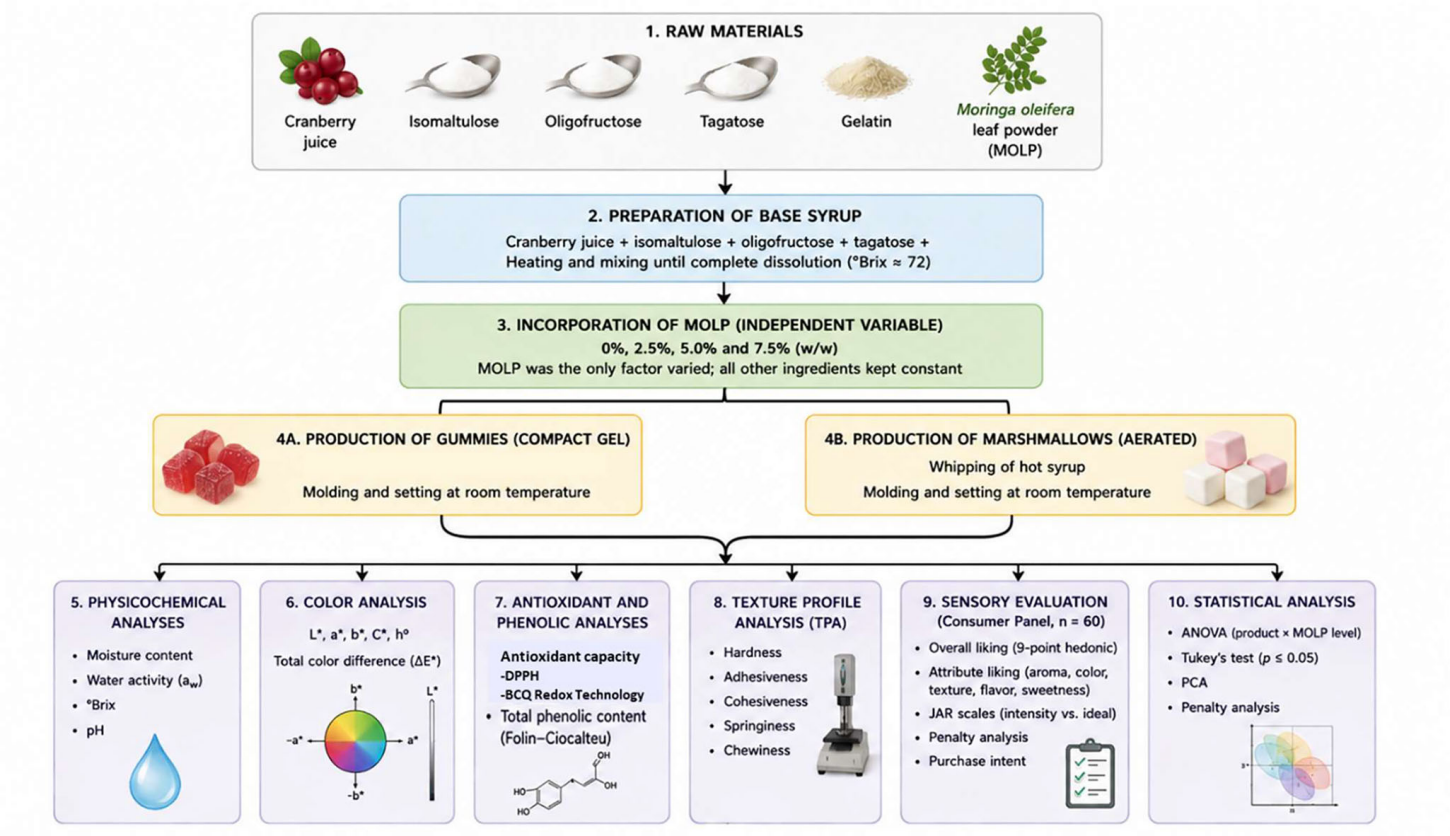
General experimental sequence followed in the study.

Figure 1 shows the means and standard deviations of the moisture content (g water/100 g candy) and water activity of two kinds of candy (marshmallows and gummies).

As shown in Figure 1, all the soft candies had an adequate moisture content, according to the recommendations for this type of product, which is 24% (Edwards and Vercet 2002), except in the case of the gummy containing 7.5% MOLP, which presented a moisture content of 27.8%.

Marshmallows presented a lower moisture content than gummies did, which can be attributed to their aerated texture characteristics. The moisture content of the marshmallows ranged from 15.8% in the control sample to 22.2% in the sample containing 7.5% moringa powder. On the other hand, gummies presented slightly higher moisture levels than did marshmallows, with the control jelly having a moisture content of 19.5%, which progressively increased to 27.8% in the sample with the highest moringa concentration. In both cases, there was a significant increase in the moisture content only when the percentage of moringa powder added was 7.5% (Shimizu and Matubayasi 2014). This increase in moisture content at higher moringa contents can be attributed to the hygroscopic nature of MOLP, which contains fibers and polar compounds capable of binding water molecules. These components increase the water‑holding capacity of the matrix, reducing the proportion of soluble solids and creating a more hydrated structure. Consequently, the availability of free water increases slightly, explaining the observed increase in a_w_ (Y. Singh and Prasad 2013).

With respect to water activity, no significant differences were detected among the marshmallow samples containing 0%, 2.5%, or 5% moringa leaf powder, with an average value of 0.7495. However, a significant increase was observed in the sample with 7.5% moringa, reaching a value of 0.81. For the gummy‐type candies, an average water activity value of 0.83 was observed for the 0%, 2.5%, and 5% formulations, with no significant differences (*p* < 0.05). A significant increase was also noted for the 7.5% formulation, reaching a value of 0.87. This finding indicates that the addition of MOLP increases the availability of water for microorganisms and chemical reactions. Nevertheless, since the water activity remains below 0.9, the growth of pathogenic bacteria in developed gummies is not possible (Chirife et al. 1996). However, molds and yeasts can grow because the water activity (a_w_) is above 0.6 (Chirife et al. 1996). Therefore, for commercial applications, additional preservation strategies, such as refrigerated storage, natural antimicrobial use, or packaging under a modified atmosphere, are needed to ensure product stability and shelf life.

The addition of MOLP to the candies decreased, in a statistically significant way, the °Brix degree of the syrup used to make the gummies (from 74.7 °Brix for the control syrup to 60.3 °Brix for the 7.5% moringa syrup). The reduction in °Brix with increasing moringa addition is due to the replacement of sweeteners and juice by moringa powder, which introduces insoluble components such as fiber and proteins (Sultana 2020; Fidyasari et al. 2024). These factors do not contribute to soluble solids, lowering the refractometric reading.

The pH of the syrups ranged between 3.40 and 4.11. These low pH values occurred because the candies were made with cranberry juice, which had pH values of 2.99 and 4.37 °Brix. A statistically significant increase in pH was observed with the addition of MOLP to the syrup (from 3.4 for the control syrup to 4.11 for the 5% and 7.5% formulations). No significant differences (*p* > 0.05) were observed between the 5% and 7.5% formulations, but they were observed with the 2.5% (pH = 3.78) formulation. The increase observed at higher moringa contents is explained by the buffering effect of moringa leaf constituents, which include minerals and organic compounds that neutralize acidity from cranberry juice, shifting the pH toward less acidic values. In general, most pathogenic bacteria, such as *Clostridium botulinum*, cannot grow in environments with a pH below 4.6 (Raatjes and Smelt 1979).

To evaluate the contribution of MOLP to the antioxidant properties of the candies, the antioxidant activity was determined via two methods, and the total phenol content is presented in Table 2. The control gummies presented an antioxidant capacity of 4.0 mg TE/100 g for the marshmallows and 3.4 mg TE/100 g for the gummies. This occurred because they were made with cranberry juice, which is very rich in antioxidants (52.11 mg Trolox/mL juice; Table 2). This already represents an improvement in nutritional content, compared with gummies made only with sugars, which do not show antioxidant activity (Periche et al. 2016). Furthermore, Table 2 shows how the addition of MOLP substantially increased the antioxidant content of the soft candies, both for marshmallows and gummies.

**TABLE 2:** Antioxidant capacity by DPPH method and BQC method and total phenol content of cranberry juice, MOLP, and candies (M: marshmallow, G: gummy).

Samples	DPPH (mg TE/100 g)	BQC (mg TE/100 g)	Total phenol content (mg GAE/100 g)
Cranberry juice	52.11 ± 0.16	796 ± 62	2.64 ± 0.4
MOLP	52.9 ± 0.4	1825 ± 33	6.32 ± 0.14
M0	4.0 ± 0.3^a^	102 ± 13^a^	0.196 ± 0.010^a^
M2.5	15.08 ± 0.16^b^	424 ± 45^b^	0.56 ± 0.3^b^
M5	26.58 ± 0.14^c^	606 ± 99^c^	0.94 ± 0.03^c^
M7.5	31.5 ± 0.3^d^	549 ± 5^bc^	0.98 ± 0.04^c^
G0	3.4 ± 0.4^A^	108 ± 19^A^	0.127 ± 0.012^A^
G2.5	14.1 ± 0.4^B^	343 ± 20^B^	0.567 ± 0.014^B^
G5	23.3 ± 0.3^C^	534 ± 10^C^	0.87 ± 0.03^C^
G7.5	29.8 ± 0.4^D^	513 ± 36^C^	0.97 ± 0.05^D^

*Note*: Different letters in the same column (lowercase letters for marshmallow and uppercase letters for gummy) indicate statistically significant differences obtained via one‐way ANOVA considering the MOLP percentage factor (0%, 2.5%, 5%, and 7.5%), with a significance level of 95%.

The total phenolic content of the control marshmallows was 0.196 mg GAE/100 g candy and that of the control gummies was 0.127 mg GAE/100 g candy, which progressively increased in both cases with the addition of moringa leaf powder, reaching 0.98 mg GAE/100 g candy for the marshmallows and 0.97 mg GAE/100 g candy for the gummies (Table 2). The higher concentration of total polyphenols in the control marshmallow‐type gummies than in the control gummy‐type samples, despite having the same proportion of sugars and cranberry juice, can be attributed to aeration. A more porous structure decreases matrix compactness, potentially limiting interactions between polyphenols and proteins or polysaccharides and facilitating their extraction during analysis (Wu et al. 2025). In addition, the distribution of cranberry juice within a less dense matrix may increase the release of phenolic compounds, and if aeration involves shorter heating times or milder temperatures, the thermal or oxidative degradation of polyphenols is minimized, contributing to greater retention (Pronina et al. 2023).

The increase in antioxidant capacity and total phenolic content with increasing MOLP level is due primarily to the high concentration of bioactive compounds, including phenolic acids, flavonoids, and vitamins, which act as radical scavengers, in *Moringa oleifera* leaves (Mishra et al. 2025; Chakraborty et al. 2025). Additionally, the base formulation contains cranberry juice, a source of polyphenols such as proanthocyanidins and anthocyanins, which already contribute to antioxidant activity even in the control samples. When moringa is incorporated, its phenolic compounds act synergistically with those from cranberry, resulting in a cumulative effect that significantly enhances the antioxidant capacity and total phenolic content. This synergy explains the progressive increase observed in both marshmallows and gummies. Although heat could reduce some heat‑labile compounds, the overall increase in antioxidant capacity and total phenolics with moringa indicates net functional retention under the present conditions, likely aided by matrix interactions (polyphenol‐protein/sugar binding) that can protect a fraction of phenolics during processing (Lapsongphon et al. 2018).

These results indicate that the addition of MOLP enhances the functional properties of gummies, so in addition to having a low glycemic index, MOLP also provides beneficial health functional properties.

Table 3 summarizes the color values (L*, a*, b*, C*, h°) of the marshmallows and gummies formulated with increasing concentrations of MOLP. Figure 3 shows an example of each candy. The incorporation of MOLP produced clear and concentration‐dependent changes in the color attributes of both the marshmallows and the gummies. In marshmallows, increasing the MOLP markedly decreased the lightness (L*) and intensified the yellow component (b*) while shifting the hue angle toward the yellow–green region. These effects reflect the dominant contribution of MOLP pigments, particularly chlorophylls and carotenoids, which display strong light‑absorbing properties and tend to mask the reddish‐violet tones provided by cranberry juice. In parallel, cranberry anthocyanins display limited stability at moderately acidic to near‑neutral pH and are prone to degradation, copigment disruption, and structural rearrangements that reduce chromatic expression, thereby favoring the prevalence of MOLP's greenish‑yellow hue (Khoo et al. 2017). The aerated, porous structure of marshmallows further amplifies these optical effects by enhancing light scattering, pigment, and matrix interactions, which can accentuate darkening and hue shifts in foam systems. In summary, the color changes were more moderate because of the denser, more homogeneous gel network, which restricted pigment dispersion and reduced the apparent chroma. Although lightness remained nearly constant, the hue angle still shifted toward the yellow–green region, indicating that even low MOLP levels alter tonality. The limited increase in chroma suggests optical dilution of MOLP pigments within the gel, whereas anthocyanins, which are often better retained in acidic, polysaccharide‑rich gels, preserve part of their color, mitigating drastic saturation changes (Enaru et al. 2021). Moreover, gel matrices and hydrocolloid environments can attenuate the oxidative and thermal degradation of chlorophylls and carotenoids relative to more open, aerated systems, thereby moderating overall color drift (A. Singh and Singh 2025). Taken together, the interplay among MOLP pigments, anthocyanin stability, and matrix architecture (foamed vs. gelled) explains both the direction and magnitude of color shifts observed across product types.

**TABLE 3:** Color values of MOLP, cranberry juice, marshmallows (M) and gummies (G).

Sample	L*	a*	b*	C*	h°	ΔE
MOLP	52.4 ± 0.4	‐ 6.11 ± 0.0	17.7 ± 0.3	18.7 ± 0.3	109.06 ± 0.09	—
Cranberry juice	16.4 ± 0.3	44.1 ± 0.4	27.8 ± 0.6	52.1 ± 0.6	32.2 ± 0.3	—
M0	86.0 ± 0.3^d^	3.5 ±0.2^c^	3.6 ± 0.3^a^	5.1 ± 0.3^a^	45.9 ± 0.7^a^	—
M2.5	74 ± 2^c^	1.0 ±0.2^b^	15.8 ± 1.7^b^	16 ± 2^b^	86.5 ± 0.6^b^	17 ± 2
M5	70 ± 3^b^	0.6 ± 0.2^a^	16 ± 4^b^	16 ± 4^b^	87.9 ± 0.9^c^	20 ± 4
M7.5	63.7 ± 1.9^a^	1.2 ±0.4^b^	19 ± 3^b^	19 ± 3^c^	86.4 ± 1.0^b^	27 ± 3
G0	24.3 ± 0.8^A^	0.31 ±0.13^A^	−0.3 ± 0.4^A^	0.61 ± 0.10^A^	306 ± 8^B^	—
G2.5	25.3 ± 0.6^B^	0.73 ±0.05^C^	2.4 ± 0.4^C^	2.5 ± 0.4^D^	73 ± 2^A^	3.0 ± 0.5
G5	24.7 ± 0.4^AB^	0.43 ± 0.09^B^	1.70 ± 0.09^B^	1.75 ± 0.09^C^	76 ± 3^A^	2.1 ± 0.1
G7.5	24.8 ± 0.3^AB^	0.38 ± 0.08^AB^	1.4 ± 0.3^B^	1.4 ± 0.3^B^	74.6 ± 1.6^A^	1.8 ± 0.3

*Note*: L*: Lightness; a*: redness to greenness; b*: yellowness to blueness; C*: chroma; h°: hue angle and color difference (ΔE).The values are the means and standard deviations (*n* = 6). Different superscript letters (lowercase letters for marshmallows and uppercase letters for gummies) in the same column indicate statistically significant differences (*p* < 0.05) obtained via ANOVA considering the percentage of MOLP (0, 2.5, 5 and 7.5) for each color parameter.

**FIGURE 2:**
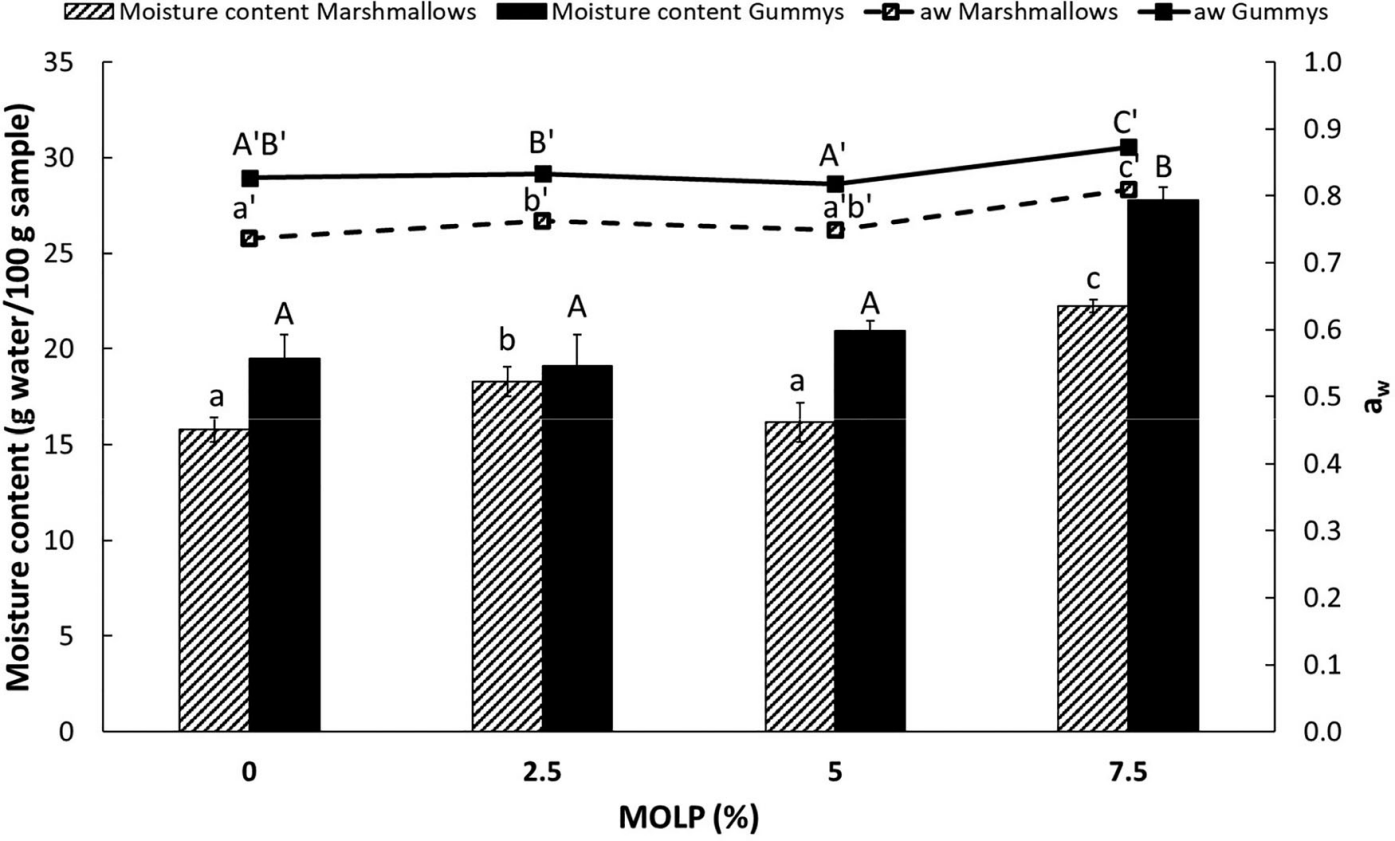
Moisture content (g water/100 g sample; bars) and water activity (a_w_) (lines) of different formulations for marshmallows and gummies enriched with different percentages of MOLP. Different letters indicate statistically significant differences (s.l. 95%) according to the factor “percentage of MOLP” (lowercase letters for marshmallows and uppercase letters for gummies).

Regarding color differences, compared to the control candies, ΔE values were much more pronounced in the marshmallows than in the gummies. In the marshmallows, a ΔE value of 17 was obtained, which increased as the percentage of MOLP increased, indicating that the color changes were noticeable to the human eye since ΔE was greater than 3 (Pathare et al. 2013). In contrast, in the case of the gummies, the color difference, compared to the control, was not as noticeable to the human eye since the ΔE values ​​were close to 2.

Table 4 shows the mean values and standard deviations ​​obtained from the TPA tests (hardness, springiness, adhesiveness, chewiness, cohesiveness, and resilience) of the different candy formulations. The TPA test allows objective analysis of the texture and replicates the chewing experience (Khan and Rahman 2021).

**TABLE 4:** Mechanical properties: Hardness (H), springiness (S), adhesiveness (A), chewiness (C), cohesiveness (Co) and resilience (R). Units are indicated in parentheses.

Samples	H (N)	S	A (N·s)	C (N)	Co	R
M0	13.1 ± 0.3^b^	9.0 ± 0.3^a^	−0.05 ± 0.03^a^	11.0 ± 0.3^b^	0.915 ± 0.003^b^	0.618 ± 0.012^a^
M2.5	8.8 ± 0.8^a^	10.0 ± 0.6^b^	−0.1 ± 0.04^a^	7.7 ± 0.9^a^	0.921 ± 0.008^b^	0.727 ± 0.005^b^
M5	15.2 ± 0.6^c^	9.9 ± 0.6^b^	−0.05 ± 0.02^a^	12.0 ± 0.6^c^	0.875 ± 0.007^a^	0.623 ± 0.006^a^
M7.5	20.2 ± 0.7^d^	9.2 ± 0.2^a^	−0.07 ± 0.05^a^	16.6 ± 0.5^d^	0.880 ± 0.006^a^	0.613 ± 0.008^a^
G0	17.7 ± 1.3^A^	9.2 ± 0.2^A^	−1.2 ± 0.7^A^	16.2 ± 1.2^A^	0.934 ± 0.009^B^	0.785 ± 0.012^D^
G2.5	30.9 ± 2.4^B^	9.0 ± 0.7^A^	−0.9 ± 0.6^A^	26.7 ± 2.6^B^	0.89 ± 0.04^B^	0.67 ± 0.03^C^
G5	59.1 ± 1.1^C^	9.0 ± 0.4^A^	−0.6 ± 0.2^A^	40.4 ± 1.0^C^	0.735 ± 0.006^A^	0.469 ± 0.009^B^
G7.5	66.7 ± 1.6^D^	9.1 ± 0.3^A^	−0.7 ± 0.6^A^	46.3 ± 1.6^D^	0.749 ± 0.009^A^	0.428 ± 0.008^A^

*Note*: The values are the means and standard deviations (*n* = 6). Different superscript letters (lowercase for marshmallows and uppercase for gummies) in the same column indicate statistically significant differences obtained via the ANOVA applied considering the factor percentage of MOLP (0, 2.5, 5 and 7.5) for each mechanical parameter, with a significance level of 95%.

The incorporation of MOLP (0%–7.5%) modulated the mechanics of two confectionery matrices with distinct architectures, marshmallows (M, aerated foams) and gummies (G, compact gels).

In summary, the progressive increase in hardness and chewiness observed as the moringa content increased is consistent with the incorporation of insoluble solids (fibers and proteins) from MOLP into the gelatinized matrix. These particles increase the effective solids content and act as fillers within the biopolymer network, limiting chain mobility and promoting a denser gel structure. Therefore, the material resists deformation more strongly (higher hardness) and requires more energy to masticate (higher chewiness). Analogous mechanical reinforcement has been reported when insoluble dietary fibers or plant particulates are incorporated into protein and polysaccharide gels, improving gel strength and water retention to a concentration threshold beyond which network continuity may deteriorate (Lv et al. 2022). The sharp increase in hardness and chewiness with increasing MOLP inclusion can be attributed to a filler–reinforcement mechanism and water redistribution within the gelatin–sugar network. MOLP produces insoluble solids (dietary fibers, proteins, and cell wall particles) that become physically embedded in the biopolymer gel, restricting chain mobility and increasing the packing density (filler effect). Concomitantly, moringa's hygroscopic components bind water, lowering the free water available for gelatin plasticization; the resulting network is less solvated and more rigid, which increases the force needed to deform the matrix (hardness) and the energy to masticate it (chewiness). These effects are consistent with the known roles of sugars/polyols and solids in gel structuring and water–biopolymer interactions (Shimizu and Matubayasi 2014; Rosenthal 2010; Sultana 2020; Peleg 2019). Under the present processing conditions, the dominant mechanisms are physical rather than chemical, including particulate reinforcement by moringa solids, competitive hydrogen bonding among gelatin, water, and added sugars, which is known to modulate solation and gelation behavior and is consistent with evidence showing that sugars alter gelatin network structure through hydrogen‐bonding competition (Avallone et al. 2022), as well as pH‐dependent network formation characteristic of physically cross‐linked gelatin systems sensitive to temperature and acidity (de Carvalho and Djabourov 1997). The texture changes are parsimoniously explained by network densification and reduced molecular mobility due to solids loading and water binding. In marshmallows, the nonlinear trajectory suggests that small additions (∼2.5%) can modify the foam microstructure, redistributing bubble sizes and lamella thickness, yielding a more elastic, less dense matrix, whereas at ≥ 5%, the filler effect dominates, and the aerated structure becomes stiffer. Related trends (strengthening or weakening depending on purity/particle size and concentration) have been documented when unrefined plant powders or pectins are added to gels (Lian et al. 2023; Zhang et al. 2023) and are consistent with the broader technological impact of *Moringa oleifera* in food matrices reported in recent reviews. The stronger effect in gummies than in marshmallows is coherent with the denser, non‑aerated structure of gummies, which transmits stress more efficiently and magnifies the filler effect; aeration in marshmallows distributes stress and partially masks the increase in firmness.

Springiness or elasticity is related to the height with which the food recovers during the time that elapses between the end of the first bite and the start of the second bite (Peleg 2019). This parameter remained essentially unchanged in gummies and varied only moderately in marshmallows, indicating that rapid recovery after primary deformation is dominated by the hydrocolloid gelling and hydration state. Moreover, the TPA literature notes that some parameters (e.g., adhesiveness and, at times, springiness) are less sensitive to solid inclusions when the gel former governs the primary elastic response (Rani et al. 2021). Adhesiveness (A), which is related to the adhesive force between food and teeth (Peleg 2019), showed no significant differences (*p* > 0.05). The values obtained for candies in this research were quite similar to those obtained by Matas‐Gil et al. (2025) (−0.154 and −0.396 N·s) in 3D‐printed gummies and significantly greater than the data obtained by Ahmad Nasir et al. (2022), who studied the texture of gummies using nutritive and nonnutritive sugars with adhesiveness of −23 N·s when candies were prepared with white sugar or −13.6 N·s with monk sugar. The low adhesiveness of sweeteners makes them appropriate for gummy formulations (Sumonsiri et al. 2021). This behavior is attributed to the hydroxyl (−OH) groups present in sugars and sugar alcohols, which can interact with water molecules around the biopolymers and affect their structure (Shimizu and Matubayasi 2014).

In contrast, cohesiveness (Co) and resilience (R) were decreased at higher doses, more noticeably in gummies, which fits mechanistic evidence showing that excess fibers/particles can increase chain separation, impede cross‑linking, and reduce reversible mobility, thereby diminishing the network's ability to recover elastic energy and maintain integrity under double compression. This has been demonstrated in protein‒fiber gels and in cases where particle size and concentration dictate the balance between reinforcement and loss of continuity (Popov et al. 2022).

Cranberry juice remains fixed in the base formulation and contributes acidity (low pH), polyphenols, and soluble solids. The acidic environment aids gelatin setting within a favorable pH window, but when moringa is added, the buffering components (minerals, proteins) from moringa slightly increase the pH, which can influence gel strength and the interaction between gelatin and sugars. In practical terms, the constant presence of cranberry provides baseline elasticity and adhesiveness; as moringa increases, the net effect shifts toward greater firmness and lower cohesiveness/resilience, particularly in the denser gummy matrix, compared with the aerated marshmallow, whose air cells scatter stress and mask part of the firmness increase.

The base used isomaltulose, oligofructose, and tagatose, each with a distinct molecular weight, hygroscopicity, and hydrogen bonding capacity. Even though their levels are held constant, they set the matrix's baseline viscoelasticity and surface stickiness. Oligofructose (short‐chain fructan) may partially behave as a soluble dietary fiber, contributing to increased viscosity and water binding capacity in confectionery systems. Likewise, isomaltulose and tagatose contain multiple hydroxyl groups capable of participating in hydrogen bonding interactions with water and gelatin, potentially influencing water mobility and structural organization within the gel matrix. Under this constant formulation background, the incorporation of MOLP likely displaces part of the soluble solids with insoluble plant particles, which may explain the decrease in °Brix and the observed reduction in adhesiveness in gummies. In addition, a reduction in the free surface water availability together with the formation of a more structured network may have contributed to the lower negative area recorded during the first compression cycle of TPA (Franck 2002; Roos and Drusch 2015; Bourne 2002).

In summary, from a technological standpoint, the data suggest that for marshmallows, a low addition (∼2.5%) favors a lighter, more elastic bite and lowers initial resistance, whereas ≥ 5% effectively increases structural firmness at the cost of lower cohesiveness at higher loadings. For gummies, MOLP levels of 5.0%–7.5% are recommended when increased hardness and cohesiveness are desired. Conversely, to preserve resilience and internal structural integrity, incorporation levels should not exceed 2.5%.

Table 5 shows the average scores for each sensory attribute of candies evaluated by the panelist on a 9‐point hedonic scale. Overall, the candies demonstrated moderate acceptability, with scores above 4.5, except for formulations containing 7.5% moringa leaf powder, which showed slight disapproval (4.14 for marshmallows and 4.08 for gummies), corresponding to the category “moderately dislike.” The control formulations, both for marshmallows and gummies, received the highest overall acceptance, with mean scores of 6.41 and 6.25, respectively, placing them between “like moderately” and “like quite a bit.” For marshmallows, the formulation containing 2.5% MOLP was the most accepted after the control, with a mean score of 5.83, ranking between “neither like nor dislike” and “like moderately.” This was followed by the 5% formulation, which scored 5.28, while the 7.5% formulation received the lowest score (4.14). Similarly, for gummies, the 5% formulation resulted in the highest acceptance after the control. This formulation was followed by the 2.5% formulation, with a score of 5.46, while the 7.5% formulation received the lowest score (4.08).

**TABLE 5:** Mean score ± standard deviation and the F‐ratio of each attribute evaluated in the samples using a 9‐point hedonic scale.

Sample	Appearance	Color	Aroma	Hardness	Gumminess	Flavor	Sweetness	Global acceptance
M0	7.3 ± 1.4	7.2 ± 1.8	6 ± 2	7.1 ± 1.4	7.1 ± 1.7	6.0 ± 1.8	6.1 ± 1.5	6.4 ± 0.9
M2.5	5.4 ± 1.7	4.9 ± 1.9	5.2 ± 1.8	7.0 ± 1.4	7.2 ± 1.9	5.8 ± 1.7	6.5 ± 1.4	5.8 ± 0.9
M5	6.1 ± 1.6	5.8 ± 1.5	5 ± 2	6.2 ± 1.7	6.3 ± 1.9	5.4 ± 1.8	5.8 ± 1.9	5.3 ± 0.9
M7.5	5.8 ± 1.8	6 ± 2	5.0 ± 1.8	6.0 ± 1.9	6 ± 2	5 ± 2	5 ± 2	4.1 ± 1.0
F‐ratio	7.71**	7.59**	1.25	3.22*	3.89*	1.63	1.62	8.51**
G0	7 ± 2	7 ± 2	6 ± 2	6.9 ± 1.6	6.6 ± 1.8	5.9 ± 1.7	6.5 ± 1.6	6.3 ± 1.1
G2.5	6 ± 2	5 ± 2	6 ± 2	6.5 ± 1.9	6 ± 2	5 ± 2	6 ± 2	5.5 ± 1.2
G5	5 ± 2	5 ± 2	5 ± 2	5.3 ± 1.9	5 ± 2	4 ± 2	5 ± 2	4.6 ± 1.3
G7.5	5 ± 2	5 ± 2	5 ± 2	5 ± 2	5 ± 2	4 ± 2	5 ± 2	4.1 ± 1.1
F‐ratio	1.96	2.85**	1.00	7.18**	4.28**	3.87*	2.68	4.94**

**Statistical significance ≥ 99% (*p*‐value ≤ 0.01). *Statistical significance ≥ 95% (*p‐*value ≤ 0.05).

From a sensory perspective, the attributes of the marshmallows most affected by the addition of MOLP were overall acceptance (F‐ratio = 8.51), appearance (F‐ratio = 7.71), and color (F‐ratio = 7.59), with a significance level of 99%. To a lesser extent, the addition of MOLP also had a significant effect (95% significance) on the hardness (F‐ratio = 3.22) and gumminess (F‐ratio = 3.89) of the marshmallows. However, the panelists did not find significant differences among the marshmallows in terms of aroma, texture, flavor, or sweetness. On the other hand, in gummy‐type candies, the addition of MOLP had a more pronounced effect on hardness (F‐ratio = 7.18), overall acceptance (F‐ratio = 4.94), and gumminess (F‐ratio = 4.28), with a significance level of 99%. The panelists also identified significant differences among the gummies in terms of color (F‐ratio = 2.85), texture (F‐ratio = 3.76), and flavor (F‐ratio = 3.87), with a significance level of 95%. The addition of MOLP did not influence the perceived sweetness or aroma of the gummies.

With respect to purchase intent, 79.31% of the panelists indicated that they would buy the control marshmallows and the M2.5% formulation if they were aware of their health benefits. Additionally, 58.6% expressed a willingness to purchase the M5% formulation, whereas 69% would consider buying the 7.5% formulation. For gummies, 75% of the tasters stated that they would purchase the control formulation, whereas 66.7% would buy the 2.5% formulation. Moreover, 50% of the panelists indicated that they would purchase the G5 formulation, and 45.8% expressed a willingness to purchase the 7.5% moringa gummy formulation.

**FIGURE 3:**
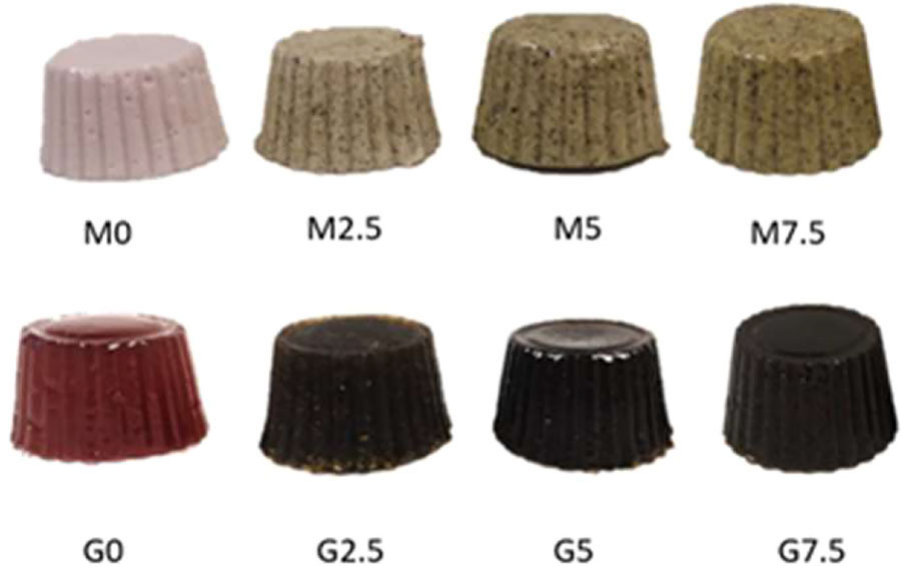
Appearance of the candies.

**FIGURE 4:**
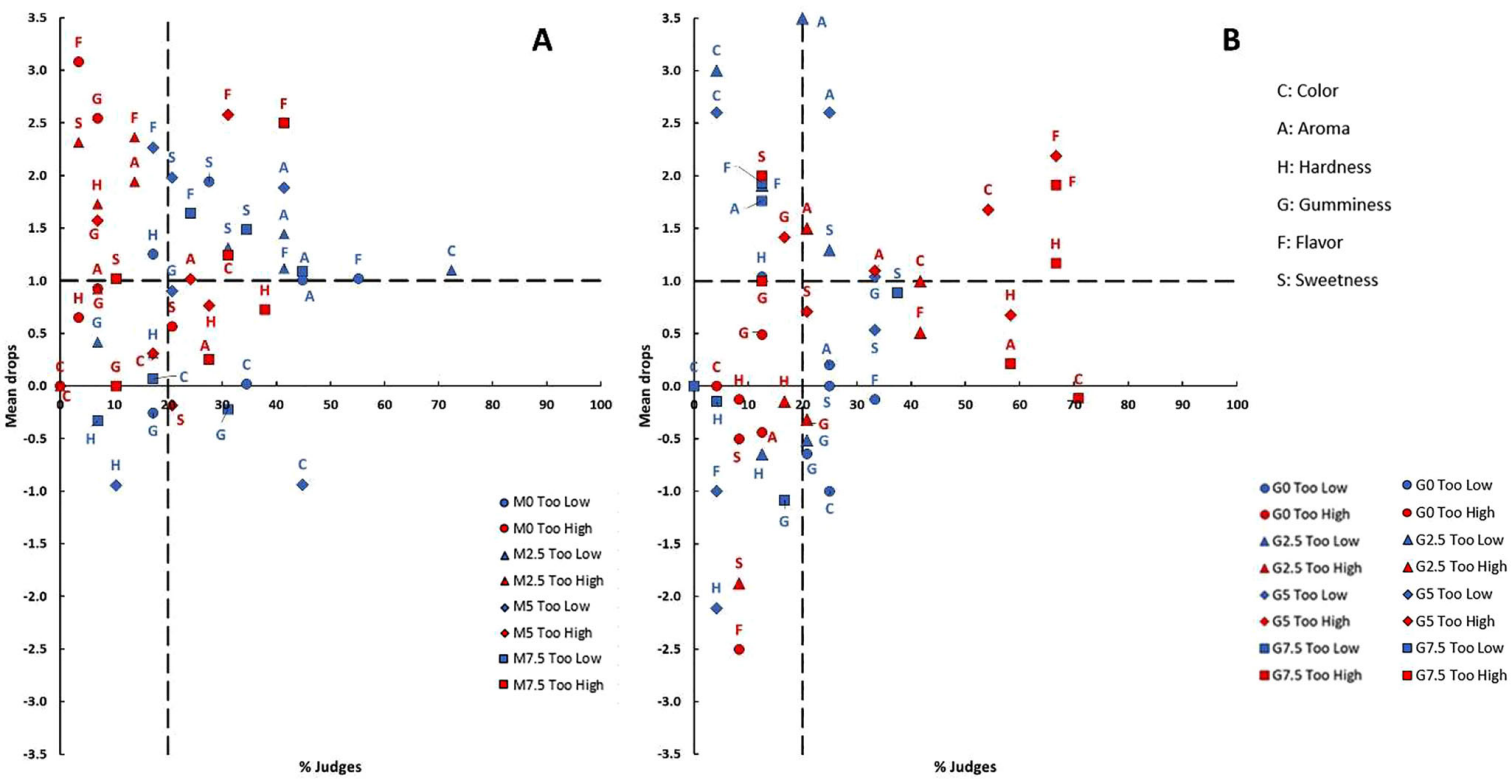
Penalty charts for marshmallows (A) and gummies (B). Blue: too low, insufficient; red: too high, excessive.

A key factor in the development of new products is sensory evaluation by consumers. JAR scales are widely used in new product development as a consumer research technique. They help determine whether the attributes present in a food product are well optimized or if their intensity needs to be increased or decreased (Ares et al. 2017; Li et al. 2014; Olegario et al. 2024). The penalty analysis chart allows for the assessment of whether judges who rated an attribute above or below the ideal point assigned a lower overall acceptance score than those who perceived the attribute as optimal. This method helps determine whether an attribute falling below or exceeding the ideal point has “penalized” the overall acceptance score. An attribute is considered to have a significant effect on overall product acceptance when the mean drop exceeds 1 and when more than 20% of the judges rate it as different from JAR. The impact of attributes on the acceptability of moringa candies was studied by a penalty chart (Ares et al. 2017; Li et al. 2014). The mean drops in acceptability based on the penalty analysis of marshmallows and gummies are shown in Figure 3, 4.

With respect to marshmallow‐type candies (Figure 4A), the most penalized attribute was flavor in the M5% and M7.5% formulations, which was rated as too intense by 31% and 41% of the judges, respectively. The flavor of the control marshmallow was penalized for being too low by 55% of the panelists. The color of the marshmallows containing 2.5% moringa was penalized by 75% of the judges since it was considered too light. This finding correlates with the instrumentally measured color value, as the control marshmallow exhibited a less intense and paler color, as shown in Figure 2. Finally, the aroma of the marshmallow formulated with 5% moringa was penalized for being too weak by 41% of the panelists.

For gummy‐type candies (Figure 4B), the most penalized attribute (mean drop = 3.5) was the aroma of the G2.5 formulation due to its low intensity; however, only 20% of participants penalized it. Flavor and hardness in the G5 and G7.5 moringa formulations were penalized (mean drop > 1) by 70% of the judges, who rated these attributes as excessively intense. Additionally, the color of the G5% formulation was penalized (mean drop > 1) by 55% of the judges for being too intense.

**FIGURE 5:**
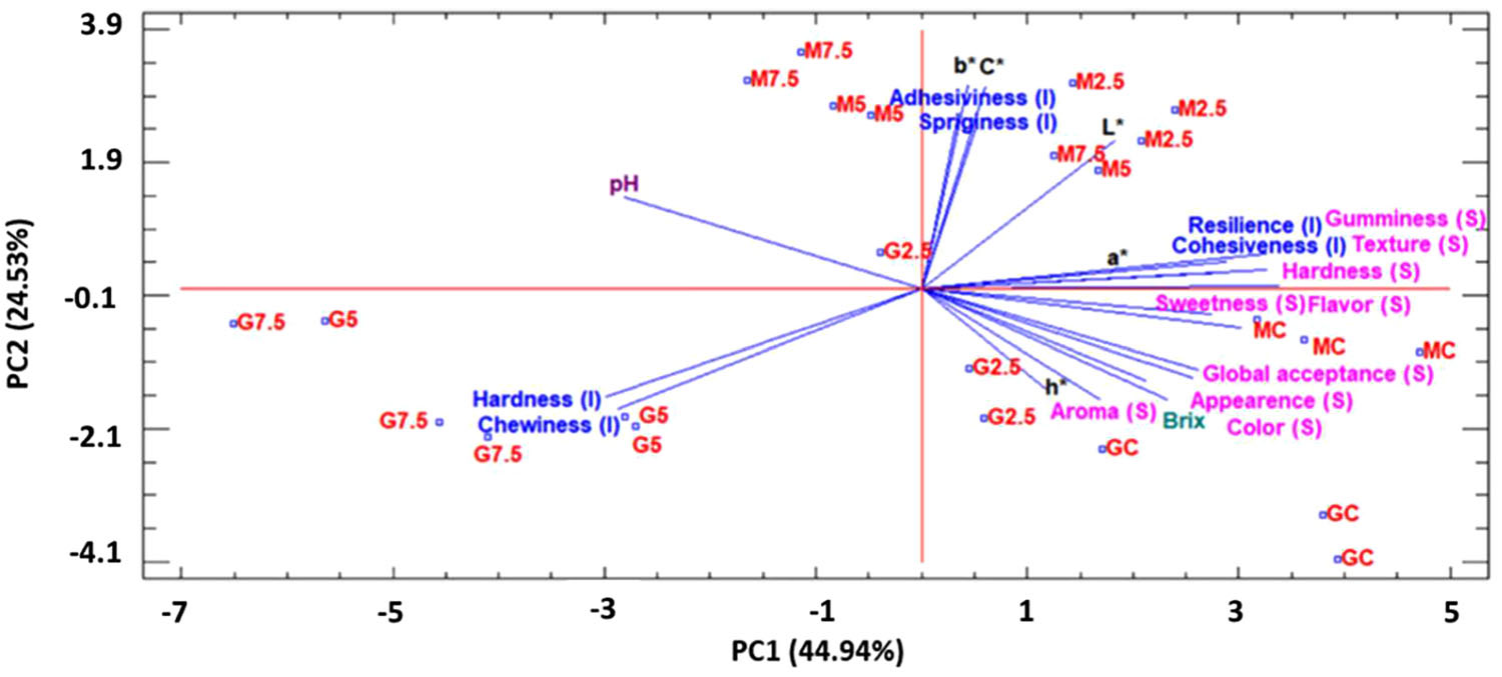
Biplot of principal component analysis (PCA) for the samples and attributes. The sample codes in red are as follows: M refers to marshmallows, G refers to gummies, and the number is the percentage of moringa powder. The sensorial attributes are shown in pink. The instrumental parameters of the texture analysis profile are shown in blue. The color parameters are shown in black. In green, ° Brix. In violet, the pH.

As previously shown by instrumental color measurements (Table 3), the incorporation of MOLP shifted the hue from the cranberry's characteristic pink toward greenish tones. These chromatic changes were reflected in consumer perception: penalty analysis revealed that marshmallows with 2.5% MOLP were rated “too light” by 75% of the panelists, whereas gummies with 5% MOLP were considered “too intense” by 55% (Figure 4). Although PCA (Figure 5) indicates that color is not the primary driver of overall liking, compared with sweetness or flavor, deviations from the expected hue negatively impact hedonic scores, particularly in dense gummy matrices where color saturation is more pronounced. These findings highlight the importance of fine‐tuning color thrugh MOLP level adjustment, aeration, or pigment balance to maintain visual appeal and consumer acceptability without compromising functional benefits.

The flavor penalization at higher MOLP concentrations is consistent with its chemical profile. The phenolic compounds and tannins present in moringa are well‐documented contributors to bitterness and astringency, whereas glucosinolates and their hydrolysis products impart pungent, mustard‐like notes that can further reduce palatability (Osakabe et al. 2024; Chan et al. 2021). Previous studies on moringa‐fortified foods consistently identify bitterness as the main barrier to consumer acceptance, and this effect intensifies as incorporation levels increase (Grosshagauer et al. 2021; Boateng et al. 2019). In addition to bitterness, high concentrations may introduce green, earthy flavors and astringent sensations due to the elevated polyphenol and chlorophyll contents, which negatively affect overall sensory perception (Osakabe et al. 2024; Grosshagauer et al. 2021). In terms of texture, the increased hardness likely resulted in a chewier sensation due to the higher solid content and reduced moisture.

The remaining attributes of the gummy formulations had mean drops below 1 and were penalized by fewer than 20% of the judges, indicating that these attributes had little influence on overall product acceptance.

**FIGURE 6:**
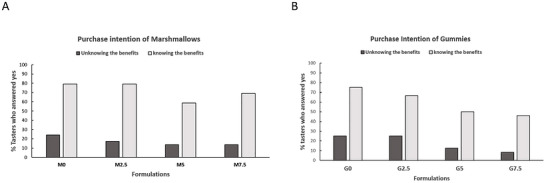
Purchase intention for marshmallows (A) and gummies (B) unknowing their benefits and knowing it.

PCA provided a comprehensive overview of the relationships between sensory attributes, instrumental texture parameters (TPA), color measurements, pH, and soluble solids content (°Brix) in the evaluated gummy samples (Figure 6). The first two principal components (PC1 and PC2) accounted for 69.47% of the total variance (44.94% and 24.53%, respectively), allowing for a reliable two‐dimensional interpretation of the data.

PC1 separated samples on the basis primarily of sensory parameters and instrumental hardness and chewiness. On the right side of PC1, sensory attributes such as sweetness, flavor, aroma, color, appearance, and overall acceptance clustered tightly together, showing strong positive correlations. These variables were strongly associated with the control samples (GC and MC), which were perceived by the panelists as having the most favorable sensory characteristics. These samples also aligned positively with °Brix, indicating that higher soluble solids content contributes positively to sweetness perception and overall acceptability.

Conversely, instrumental parameters such as hardness (I) and chewiness (I) were located on the negative side of PC1 and were strongly associated with samples G5 and G7.5. These samples exhibited high instrumental hardness and chewiness and received low sensory ratings, highlighting a potential negative relationship between instrumental firmness and consumer preference. This divergence suggests that excessive firmness, although objectively measurable, may not be desirable from a sensory standpoint.

The color parameters (L*, a*, b*, C*) were mostly associated with samples M2.5 and M5 and aligned moderately with instrumental adhesiveness and springiness. These samples did not cluster closely with the most highly rated sensory attributes, suggesting that while color contributes to visual appeal, it may not be the primary driver of overall consumer liking in this product matrix.

Notably, the variable pH was positioned away from the main cluster of variables, indicating a limited correlation with both the instrumental and sensory data. In contrast, °Brix strongly aligns with sensory sweetness and acceptability, reinforcing the role of sugar content in determining palatability.

Overall, the PCA biplot revealed a clear separation between formulations that were highly rated sensorially (e.g., GC and MC) and those with less favorable sensory profiles (e.g., G5 and G7.5), which were instead characterized by higher instrumental hardness. This suggests that while instrumental texture analysis is useful for understanding product mechanics, sensory evaluation remains essential for predicting consumer acceptance.

The highest sensory acceptance observed for the 2.5% MOLP formulation can be attributed to its ability to maintain desirable color, flavor, and texture while avoiding the bitterness, astringency, and excessive hardness associated with higher MOLP levels. Although its functional benefits were lower than those of the 5% or 7.5% extracts, consumer liking was influenced primarily by hedonic attributes rather than by antioxidant capacity or phenolic content. These findings, supported by penalty analysis and PCA, indicate that moderate MOLP addition achieves an optimal compromise between sensory quality and functional enhancement.

To balance health benefits with sensory appeal, formulators should adopt a compromise strategy that combines moderate MOLP inclusion with technological adjustments such as aeration (for marshmallows), particle size control, and pairing moringa with naturally sweet or fruity components such as cranberry juice to mask off‐flavors and maintain visual appeal. Within this framework, the range of 2.5%–5% MOLP emerges as optimal, offering a practical pathway to maximize both nutritional value and market acceptance.

As can be seen in Figure 5, the tasters' purchase intention was initially low when they were unaware of the health benefits of both types of gummies (≈17.2% for marshmallows and ≈17.7% for gummies, on average). However, after being asked the question and informed of the health benefits of these confectioneries, purchase intention rose to 71.6% for marshmallows and 59.4% for gummies. Besides, the higher the moringa level addition, the lower the purchase intention, especially in gummies.

Penalty analysis and PCA confirm that while sensory attributes such as flavor and texture remain critical, the communication of health‐related advantages strongly influences consumer willingness to buy. Leveraging these factors in marketing can be achieved through clear labeling of antioxidant content, low‐glycemic sweeteners, and natural colorants, combined with clean‐label positioning and storytelling that emphasize wellness and sustainability. This strategy reinforces the product's dual appeal, nutritional improvement and enjoyable sensory experience, maximizing its potential in health‐conscious markets.

## 4 Conclusion

This study demonstrated that incorporating MOLP into soft candies formulated with low‐glycemic sweeteners and cranberry juice significantly increased the antioxidant capacity and total phenolic content of these materials, contributing to their functional value. However, sensory evaluation revealed that consumer acceptance declines at higher MOLP levels owing to increased bitterness and firmness, highlighting the need for a balanced approach. The formulations containing 2.5%–5% MOLP achieved the best compromise between nutritional improvement and sensory quality, with 2.5% providing the highest liking and 5% providing greater functional benefits. Penalty analysis and PCA confirmed that sensory attributes such as flavor, texture, and color remain the primary drivers of acceptance, whereas health‐related claims strongly influence purchase intent (79.3%). These findings recommend that moderate MOLP inclusion, combined with technological strategies such as aeration and flavor masking, offers a practical pathway for developing confectionery products that align with consumer preferences and health trends.

### 4.1 Limitations and Future Work

The present article prioritizes technological performance (color, texture profile, a_w_/°Brix/pH) and consumer response under a fixed cranberry–low‐glycemic sweetener matrix, with MOLP as the only variable factor. In follow‐up work, we will complement these data with composition analyses: (i) mineral profile; (ii) amino acid quantification; and (iii) phenolic subclass/secondary metabolite analyses of both ingredients (MOLP, cranberry juice) and finished products across MOLP levels. These analyses, together with in vitro digestion/bioaccessibility already planned, will deepen the interpretation of the observed texture–color–hedonic trade‐offs and support composition‐linked claims. While the total phenolic content and antioxidant activity of the methanolic extracts were determined, future studies will incorporate in vitro simulated oral–gastro–intestinal digestion to evaluate the bioaccessibility and retention of functional components (e.g., polyphenols from moringa and cranberry) after digestion, as well as potential matrix effects on their stability and release. These data provide a more clinically relevant understanding of the functional value of the developed candies.

Because marshmallows represent an aerated confectionery matrix, the present findings may also be relevant for other low‐density confectionery products. However, extrapolation should be performed cautiously because aerated systems present distinct structural and rheological behaviors, compared with compact gel matrices such as gummies.

Furthermore, although the health benefits of the sweeteners used are supported by previous scientific evidence, future studies should incorporate in vitro antidiabetic assays to validate the potential glycemic control effects of these formulations.

## Author Contributions

**Lourdes Cervera‐Chiner**: conceptualization, methodology, formal analysis, investigation, writing – original draft, writing – review and editing, data curation. **María Luisa Castelló**: conceptualization, methodology, investigation, writing – review and editing, resources, project administration, funding acquisition, supervision. **Francisco José García‐Mares**: conceptualization; methodology; writing – original draft; resources. **María Dolores Ortolá**: conceptualization, methodology; writing – review and editing, funding acquisition, resources, supervision, project administration, investigation.

## Conflicts of Interest

The authors declare no conflicts of interest.
